# Accelerated reliability testing of Cu-Al bimetallic contact by a micropattern corrosion testing platform for wire bond device application

**DOI:** 10.1016/j.mex.2021.101320

**Published:** 2021-03-26

**Authors:** Goutham Issac Ashok Kumar, John Alptekin, Joshua Caperton, Ashish Salunke, Oliver Chyan

**Affiliations:** Interfacial Electrochemistry and Materials Research Lab, Department of Chemistry, University of North Texas, Denton, TX 76203-5017, United States

**Keywords:** Packaging reliability, Wire bonding failures, Galvanic corrosion, Bimetallic contact

## Abstract

Accelerated reliability testing of integrated circuit (IC) packages, such as wire-bonded devices, is a useful tool for predicting the lifetime corrosion behavior of real-world devices. Standard tests, such as highly accelerated stress test, involves subjecting an encapsulated device to high levels of humidity and high temperature (commonly 85–121 ⁰C and 85–100% relative humidity). A major drawback of current reliability tests is that mechanistic information of what occurs between *t* = 0 and device failure is not captured. A novel method of in-situ investigation of the device corrosion process was developed to capture the real time mechanistic information not obtained in standard reliability testing [1]. The simple, yet effective methodology involves:

• Immersing a micropattern or device directly into contaminant-spiked aqueous solution, and observing its morphological changes under optical microscope paired with a camera.
• Short (2–48 h) time required for testing (compared to 24–300 h of standard tests).
• No need for humidity chambers.

## Graphical abstract

**Figure:**
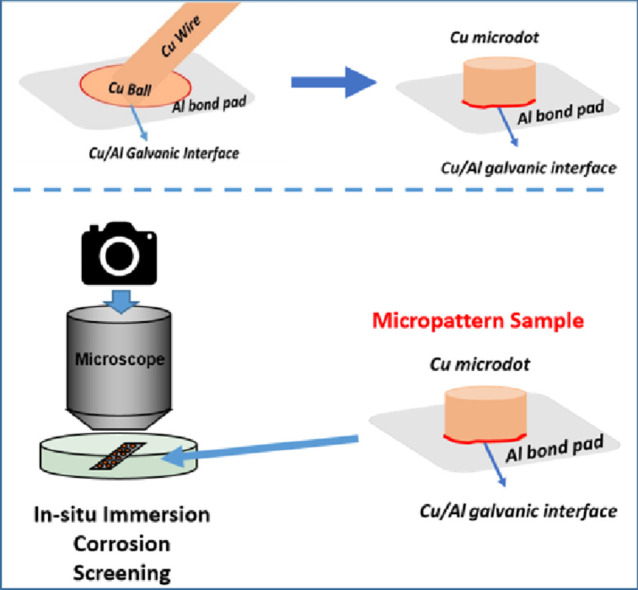

**Table:** Specifications table

Subject Area	Materials Science
More specific subject area	*Microelectronics Reliability Testing*
Method name	*Direct Immersion Corrosion Screening of Micropattern Corrosion Testing Platform for IC Device Corrosion Research*
Name and reference of original method	*Micro-pattern Corrosion Screening on Bimetallic Corrosion for Microelectronic Application.*https://doi.org/10.1016/j.electacta.2016.05.189*[**1**]*
Resource availability	*N/A*

## Method details

### Background/Motivation

The integrated circuits (ICs) industry is tightening its standards to near-zero ppb defects due to the need for safety in autonomous vehicles and reliability of wearable electronics [2]. The all-terrain and always-on operational conditions of these devices leads to greater susceptibility to corrosion through the exposure of halide contaminants from the surrounding environment. The conventional tests for evaluating ICs include biased and unbiased highly accelerated stress tests (HAST) in which devices are subjected to conditions of 85–121 ⁰C and 85–100% relative humidity from 24–300 h. These tests require the use of specially built chambers to maintain the constant temperature and humidity required to stress the devices. After being subjected to HAST testing, devices are typically evaluated “post-mortem” by measuring their change in electrical connectivity, along with imaging after removing molding compound [3,4].

**Fig. 1:**
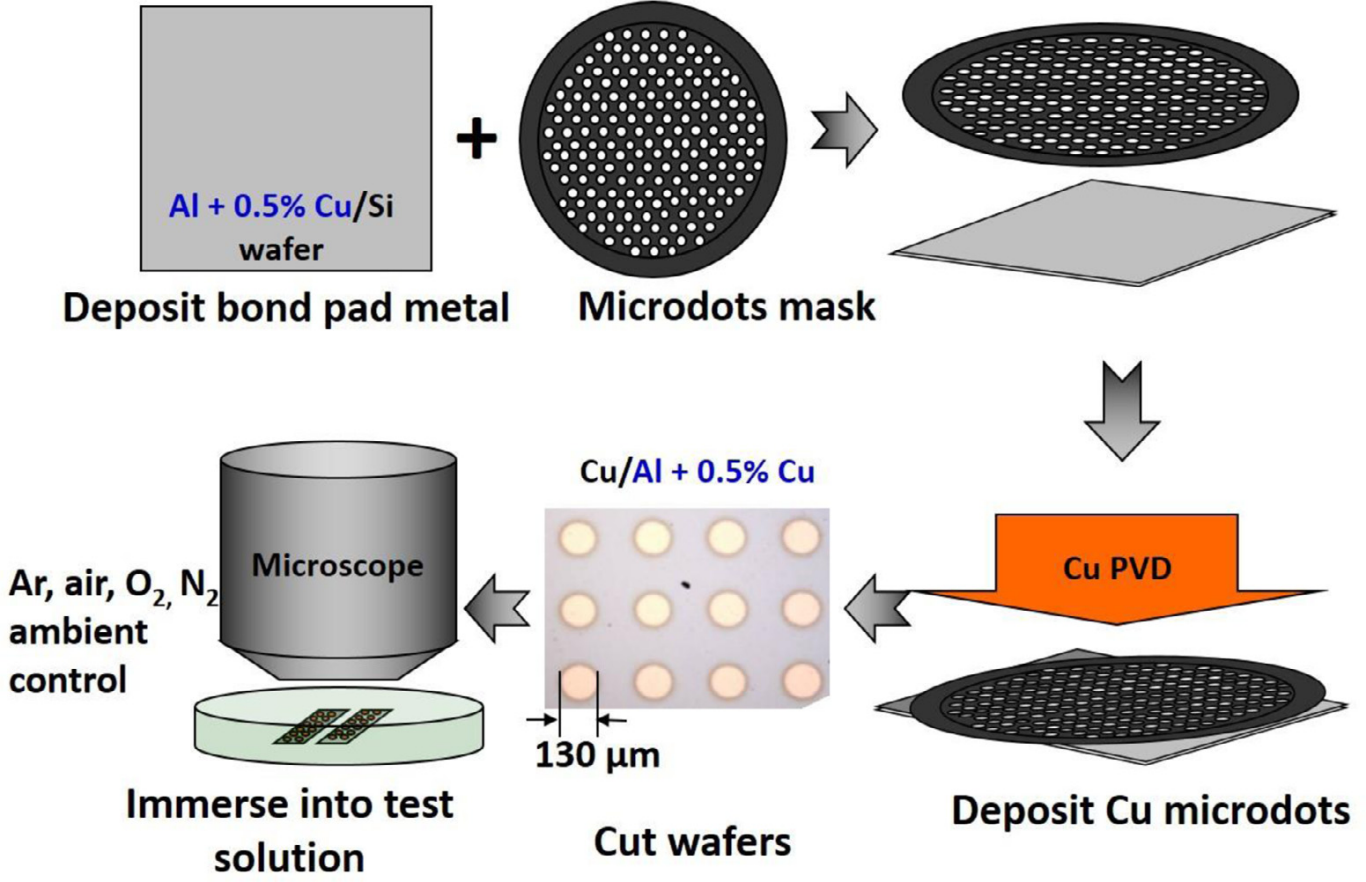
Graphical representation of microdot platform construction steps and testing.

Conventional reliability testing of wire bonded devices (WBDs) is a costly process because it requires the sacrifice of manufactured devices. The testing can take upwards of 300 h to complete, leading to long wait times for researchers to gather data. Furthermore, the high humidity and temperature conditions within HAST and bHAST chambers introduce difficulties in observing the microscopic morphological changes as they occur within the device structure. To supplement conventional reliability testing and complement its drawbacks, a new technique of direct immersion screening was devised [1]. It is important to also note the differences between the conditions of standard bHAST testing and the immersion screening method described, as they may result in different kinetically favored corrosion mechanisms. These differences are the higher temperature, humidity and applied bias in bHAST vs the full immersion into room temperature liquid in the described work. Investigation is carried out by immersing a device or micropattern (simulated device) within a small container containing a contaminant-spiked aqueous solution, and recording its morphological changes using a digital camera-mounted microscope. Here, the critical failure interface (the peripheral contact between copper (Cu) wire and aluminum (Al) bond pad) of a copper WBD was simulated using a magnetron plasma vapor deposition (PVD) scheme. Blanket Al + 0.5% Cu, serving as the “bond pad”, was sputtered onto silicon wafer. Cu microdots, serving as the “wire bond”, were then sputtered onto the simulated bond pad to establish the peripheral bimetallic contact, as illustrated in Fig. 1. The pattern of microdots sputtered creates hundreds of “wire bond” interfaces within a few square centimeters of wafer used. The corrosion of the simulated devices was observed under high resolution optical microscope while immersed in a 5–20 ppm chloride solution at pH 5 (to simulate the contaminant from epoxy molding compound leading to actual corrosion of the WBD [5]) and was found to closely resemble the corrosion of wire bonds of real copper wire bonded devices under same immersion conditions. By using this rapid and less costly technique, the authors had a faster turn-around time for determining a major corrosion pathway in Cu WBDs, and allowed for testing various corrosion inhibitors and inhibitor application methods that demonstrated corrosion protection when applied to real copper WBDs. The micropattern screening technique can be used as a cost-effective platform for exploration of various metal interfaces and corrosion prevention studies for WBDs and other ICs.

### Required reagents and equipment

• Optical microscope with top-down sample illumination, and digital image capture capabilities (minimum magnification of 100x).
• Computer with connections and software to capture video obtained from microscope system.
• Laboratory grade sodium chloride and sulfuric acid.
• Ultra-pure water (Resistivity = 18.1 MΩ) acquired from a water purification system.
• Packaged wire-bond or other integrated circuit devices without epoxy molding or encapsulation.
• Petri dishes (no gel), or other clean, small size containers to hold testing solution and sample.
• Magnetron sputtering unit, along with appropriate metal targets for sputtering (in this specific case, 99.99% Cu, and Al with 0.5% (wt.) Cu.
• Silicon wafer substrates to sputter upon.
• Stainless steel microdot mask template (dot diameter 100–200 µm, dot spacing also 100–200 µm, mask thickness 0.1 mm–1 mm) recommended hole area / overall area ratio of approximately 0.3.

### Procedures

#### Immersion screening of wirebonded device

1. Prepare a solution of 5–20 ppm Cl⁻, and adjust to pH 5 using sulfuric acid (monitor using pH probe). Note: water used to prepare solution should be ultra-pure (Resistivity = 18.1 MΩ)
2. Cut the desired packaged device from its leadframe using a pair of clean scissors in a contaminant-free environment.
3. Place device in a clean petri dish (cover if transporting to different room for testing).
4. Place device, still in the petri dish, under the microscope objective (remove petri dish cover if used). Bring microstructures into focus, and adjust microscope exposure and gain until the device is clearly viewed.
5. Begin video recording using the microscope camera software, then add 5–10 mL of the 5–20 ppm Cl⁻ pH 5 solution to the petri dish.

Note: Ensure the video starts recording before adding solution. After adding solution, the microscope focus may need to be adjusted.

#### Immersion screening using microdot platform

1. Deposit bond pad material (in this case, Al with 0.5% Cu) onto silicon wafer using magnetron sputtering. Sputtered metal should match thickness of actual bond pad (this case was 2–4 µm) [6].
2. On the same wafer, place the microdot mask gently on the surface, and sputter wire material (in this case, pure copper) up to 50–200 nm thickness. Make sure the microdot mask does not move once placed to prevent microscopic scratches to the wafer.
3. After sputtering, cut 1 × 1 cm² squares of the wafer for immersion screening, and store in a clean petri dish with a cover as needed.
4. For immersion screening, place a 1 × 1 cm² wafer square into a clean petri dish, and place under the microscope objective lens.
5. Bring the sample into focus, add immersion screening solution, and then start video recording [important: enable frame-by-frame time stamp recording for later video analysis]. If desired, and if your software allows, setup time dependent image capturing (ex: every 1 s, or every 1 min) to save hard drive space when capturing slower processes.

#### Data analysis of timelapse video

1. Initially watch the video in fast-forward, noting times of important sample surface changes (such as pitting, undercutting, surface oxidation, dendrite formation, and gas evolution).
2. Using the video time-stamp, determine the amount of time for event initiation (eg. what time the first point of gas evolution was observed). The amount of time to corrosion or gas initiation can be qualitatively correlated to how strongly reactive the galvanic system is. This should, however, be verified with electroanalytical techniques (eg. Zero Resistance Ammetry).
3. Follow-up on the noted times of sample change, and observe the morphology change one frame at a time, to note down important details (eg. where does dendrite growth initiate, and is there any accompanying phenomena such as gas evolution?)
4. With high quality imaging, further analysis can be possible by digital zoom on each frame. This can be helpful for examining the boundary between the deposited metal dot and the substrate.

### Method validation

**Fig. 2:**
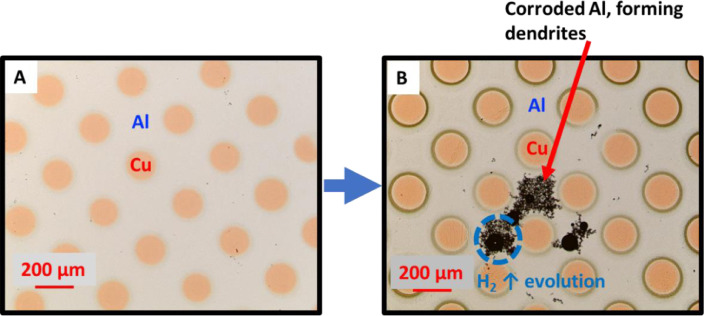
**A.** Before immersion and **B.** After 2 h of immersion of the microdot pattern immersion screening (Cu dot on blanket Al + 0.5% Cu) in 5 ppm chloride at pH 5.

**Fig. 3:**
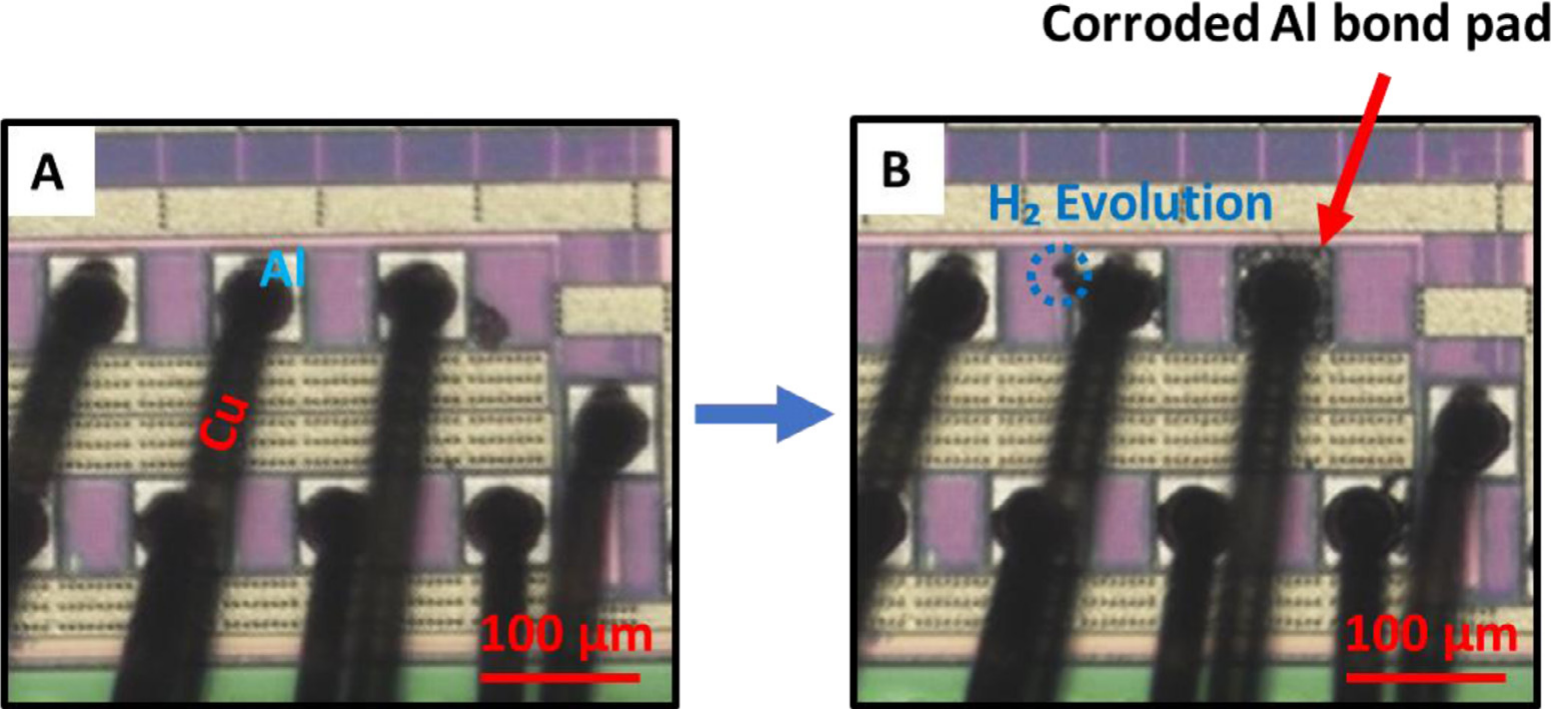
Real Cu WBD corrosion screening in 20 ppm chloride at pH 5. *A*. before immersion and *B*. After 1 h of immersion.

**Fig. 4:**
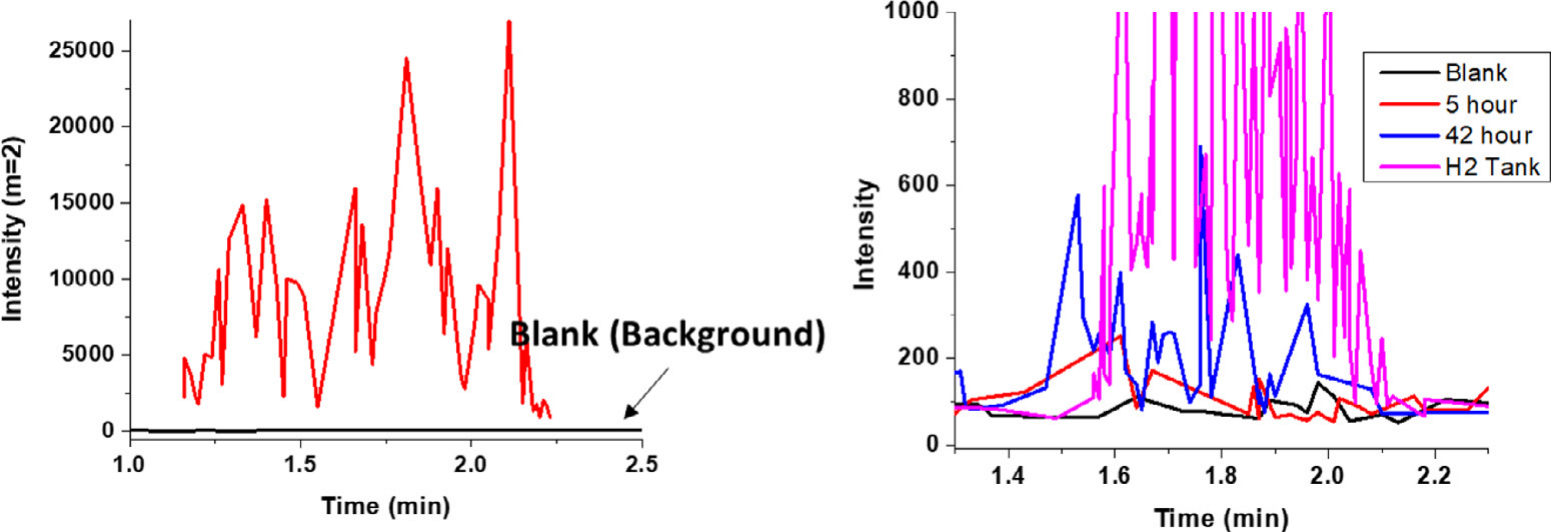
GC-MS monitoring signal at m/z = 2 of gas evolved from corroding samples while immersed in chloride solution.

As seen in Figs. 2 and 3, the corrosion around the simulated and real bond pads follow the same general mechanism. There was a dendrite formation that extended from the Cu/Al interface and propagated through the bond pad. This was accompanied by hydrogen gas evolution (confirmed by GC-MS, in Fig. 4) [7]. Since the corrosion mechanism of the microdot platform replicates that of the real wire bond device in immersion screenings, the microdots can be used in place of real devices when exploring the use of different corrosion inhibitors and the effects of different contaminants within solution.

## Declaration of Competing Interest

The authors declare that they have no known competing financial interests or personal relationships that could have appeared to influence the work reported in this paper.
